# Patient perceptions and preferences of minimally invasive treatment modalities in varicose veins: a cross-sectional survey

**DOI:** 10.3389/fcvm.2024.1382764

**Published:** 2024-04-25

**Authors:** Qian Li, Xiaotao Wang, Bin Meng, Xinle Chen, Mingmin Xu

**Affiliations:** ^1^ UItrasonic Diagnosis Department, Zhejiang Rongjun Hospital, Jiaxing, Zhejiang, China; ^2^ Neurosurgery Department, Zhejiang Rongjun Hospital, Jiaxing, Zhejiang, China

**Keywords:** varicose veins, minimally invasive therapies, patient preferences, treatment perceptions, vascular surgery

## Abstract

**Purpose:**

Minimally invasive therapies (MIT) have gained popularity due to their capacity to reduce trauma, enhance aesthetic outcomes, and shorten recovery periods. This article explores patients’ perceptions and preferences regarding MIT for varicose veins (VVs) while analyzing associated influencing factors to provide a better understanding.

**Patients and methods:**

A cross-sectional survey at Zhejiang Rongjun Hospital was performed from January 2022 to June 2023, involving 305 participants with VVs. The questionnaire assessed patient demographics, VVs severity, prior treatment experiences, and treatment preferences. Statistical analyses, including chi-square and Kruskal-Wallis tests, were conducted to explore the correlations between patient characteristics, treatment preferences, and factors influencing these choices.

**Results:**

Nearly half of the participants (44.3%) lacked information on any surgical options, whereas a slight majority (55.7%) possessed familiarity with at least one treatment modality, and only 9.8% knew of all six treatment methods presented. Patient surveys discerned that the majority (68.5%) declared an inadequate grasp of treatment methodologies to articulate a treatment preference. Among the 96 patients who made a treatment choice, 24.0% opted for traditional surgery, while 76.0% chose MIT and a higher preference for MIT among male patients compared to female patients (*p* = 0.006). The patients preferred treatment options for VVs significantly affected by vascular surgeon recommendations and the number of follow-up visits (r = 0.129, *p* = 0.024; r = 0.122, *p* = 0.033).

**Conclusion:**

The study highlights limited awareness of MIT among Chinese patients with VVs. The insights emphasize the influential role of vascular surgeons’ recommendations and suggest a growing predilection for less invasive treatments due to their advantages in recovery and aesthetics. Provider-patient communication, including education about available treatments and shared decision-making, is essential to align treatment plans with patient expectations and improve outcomes.

## Introduction

Varicose veins (VVs) are a common manifestation of chronic venous disease, characterized by the winding and expansion of superficial veins in the lower legs, commonly accompanied by pain, heaviness, itching, swelling, and pulsating discomfort (1). In developing countries, the prevalence of VVs is lower than that in developed countries (2–4). In Western nations, the prevalence of VVs is 10%–15% in males and 20%–25% in females (3, 5). China has demonstrated a prevalence rate of 8.39% for VVs (6). The presence of VVs significantly affects patients’ quality of life, giving rise to multiple complications such as ulcers, bleeding, thrombosis, and cosmetic concerns, leading to considerable inconvenience and distress for patients (1). Currently, the treatment methods for VVs are varied, mainly classified as pharmacological, compression-based, sclerotherapy, and surgical procedures (7).

Surgical treatments are usually classified based on the degree of trauma they cause: traditional surgical modalities and Minimally invasive therapies (MIT). Traditional surgical modalities include high ligation of the saphenofemoral junction (SFJ), the great saphenous vein (GSV) stripping and phlebectomy. MIT refers to procedures such as endovenous mechanochemical ablation (EMCA), radiofrequency ablation (RFA), endovenous laser ablation (EVLA), and ultrasound-guided foam sclerotherapy (UGFS) (8–11). These MIT have consistently been reported to reduce trauma, improve aesthetic outcomes, and expedite recovery compared to traditional surgical modalities. Although multiple studies have demonstrated the long-term efficacy of MIT (12, 13), additional research is necessary to confirm their sustained effectiveness (10, 14).

While significant progress has been made in technology related to treatment modalities for chronic disease patients, it is recognized that understanding patients’ perspectives and preferences is crucial for optimizing treatment decisions and outcomes (15–17). Previous research indicates that improved patient understanding of management protocols for chronic diseases can lead to better adherence and ultimately result in improved health outcomes (18, 19). Therefore, investigating patients’ perceptions and preferences regarding these MIT procedures for VVs is paramount. This article focuses on patients’ perceptions and preferences for MIT procedures for VVs, exploring their awareness, attitudes, and preferences and analyzing the influencing factors. Through gaining in-depth insight into patients’ viewpoints, healthcare professionals and researchers can tailor treatments, strengthen patient education, and advance varicose vein management, ultimately enhancing patients’ medical outcomes.

## Material and methods

### Study design

The survey instrument was structured into three sections and was developed based on prior study methodologies (20). The initial section of the questionnaire is designed to collect demographic data from the study participants. The second section probes into the severity of the participants’ VVs and their previous treatment experiences, segmenting into three dimensions with 13 items, while the third section delves into participants’ treatment preferences, organized into four dimensions with 20 items. The survey originated in English was translated into Chinese and then back-translated into English by two bilingual research personnel to maintain semantic integrity. The questionnaire was found to have acceptable internal reliability with a Cronbach α coefficient of 0.75.

The sample size was defined according to Hatcher (1994), who recommended that the number of subjects should be 5 times the number of variables (equivalently a 5:1 subject-to-variable ratio), with a minimum of 100 subjects (21). Nonetheless, some researchers suggest that the optimal sample size should be established according to a subject-to-variable ratio of 10:1 (22). Based on this premise, the 33 items in this survey would require 330 samples under the best sample design. Assuming no response bias, the sample size should be augmented by 10%. Hence, the ideal sample size would be 363.

Ethical clearance for this research was granted by the Zhejiang Rongjun Hospital Ethics Committee, documented by Approval No: 2022-LSY-10. Moreover, it conformed to the Helsinki Declaration principles, guaranteeing that informed consent was obtained from all participants.

### Participant recruitment and selection

The investigation spanned from January 2022 to June 2023 at Zhejiang Rongjun Hospital. Investigative scholars thoroughly examined referral documents from general practitioners, centering attention on patients consistently referred to a surgeon with expertise in VVs treatment. Invited selected patients partook in these anonymized questionnaires preceding their engagement with the vascular surgeon. It is paramount to note that investigators abstained from imparting knowledge to participants before questionnaire completion to preserve response objectivity. The employed research design and subsequent data analysis proficiently amassed and synthesized information on determinants influencing varicose vein treatment decisions.

The criteria for participant inclusion in the study consisted of the following: (1) Patients with thigh and leg varies resulting from major saphenous vein valve incontinence and saphenous trunk reflux leading to phlebo-lymphoedema. (2) Age over 18 years. (3) Proficiency in reading and speaking Chinese, as well as providing informed consent. Exclusion criteria included patients with only telangiectatic or reticular veins, active leg ulceration, current or previous deep vein thrombosis or occlusion, deep venous reflux, and a history of deep vein thrombosis, pulmonary embolism, or stroke. Additionally, study participation was conducted voluntarily without financial compensation.

### Data analysis

The present study utilized a stringent random sampling technique to conduct the survey. After that, the survey questionnaires were issued, and the accumulated raw data were diligently recorded in an Excel spreadsheet. Data analysis was performed using SPSS statistical software (version 20.0, IBM SPSS Inc, Chicago, IL, USA). Descriptive statistical measures, such as frequency and percentage, were employed to encapsulate the socio-demographic profiles of participants along with their responses to varied questions. The Kruskal-Wallis test was applied to establish correlations between socio-demographic factors and the severity of varicose vein symptoms. Associations between demographic factors and patient preferences were investigated using either the chi-square test or the Kruskal-Wallis test. A test level of α = 0.05 was set, and *p* ≤ 0.05 indicated statistically significant differences.

## Results

### Participant characteristics

Of the 363 eligible participants afflicted with VVs invited to this study, 305 patients (an 84.0% response rate) completed the questionnaire. Among these 305 participants, females constituted 57.4%. Pertaining to the age demographic, 59.7% of patients were categorized within the 31–40 years bracket. Additionally, nearly half of the patients, 49.5%, possessed a university-level education. Regarding occupational status, 25.6% served in corporate roles, 20.3% were self-employed professionals, and 20.7% were in healthcare professions. Further, no occupation, embodying the unemployed, homemakers, students, or the retired, accounted for 13.4%. Public sector employment, including civil servants, educators, law enforcement, or military personnel, comprised 12.5%, and farmers 7.5%. Hypertension was the most prevalent pre-existing chronic condition, representing 8.5%. For an extensive demographic breakdown, refer to Table 1.

**Table 1:** Demographic characteristics of study population (*n* = 305).

Characteristics	No. of patients (%)
Sex	
Men	130 (42.6%)
Women	175 (57.4%)
Age range (Years)	
≤30	36 (11.8%)
31–40	182 (59.7%)
41–50	58 (19.0%)
51–60	14 (4.6%)
>60	15 (4.9%)
Education	
Master's degree/PhD	18 (5.9%)
Bachelor's degree	151 (49.5%)
High school/technical secondary school	72 (23.6%)
Junior middle school	50 (16.4%)
Below junior middle school	14 (4.6%)
Employment	
Awaiting job assignment	33 (10.8%)
Part-time	20 (6.6%)
Full time	234 (76.7%)
Retire	18 (5.9%)
Occupations	
Farmers	23 (7.5%)
Public sector employee	38 (12.5%)
Private-sector employees	78 (25.6%)
Medical staff	63 (20.7%)
Self-employed workers	62 (20.3%)
No occupation	41 (13.4%)
Prior history of chronic diseases	
Diabetes	9 (3.0%)
Previous heart attack/heart failure/angina	6 (2.0%)
Chronic breathing problems	7 (2.3%)
Asthma	4 (1.3%)
Epilepsy	1 (0.3%)
High blood pressure	26 (8.5%)
Previous blood clots in the leg or lung	3 (1.0%)
Previous stroke or mini-stroke	2 (0.7%)

Public sector employees included civil servants, teachers, police, and military; no occupation included students, homemakers, or retired and unemployed residents.

### Participants’ reasons for seeking treatment and their prior treatment experience

**Figure 1:**
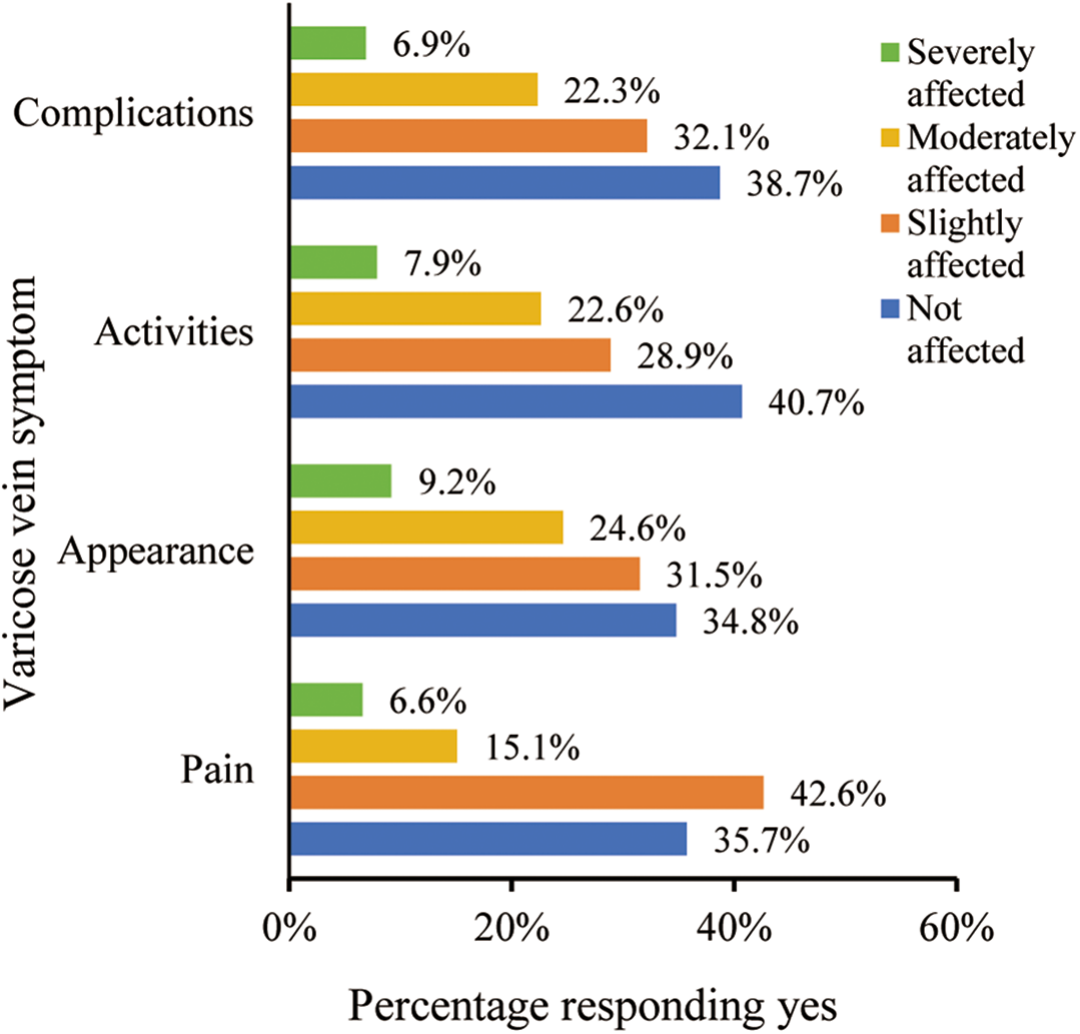
Distribution of varicose vein symptom severity in the participants.

Of the 305 respondents, 21.7% reported experiencing moderate to severe pain, while 33.8% indicated significant cosmetic concerns. Moreover, 30.5% experienced substantial mobility limitations, mirroring those with moderate to severe complications (Figure 1). The analysis indicated a correlation between patients’ socio-demographic characteristics and the intensity of symptoms. Patients with lower education levels exhibited a higher frequency of complications, whereas no significant associations were identified with gender, age, or occupation (Table 2). Among the participants, 23 individuals (7.5%, 23/305) had received treatments related to varicose veins 13 patients received compression therapy, while 10 patients received medication treatment. Of these 23 patients, a majority of 65.2% (15/23) conveyed satisfaction or high satisfaction with prior treatment results, 26.1% (6/23) remained neutral, and 8.7% (2/23) articulated dissatisfaction.

**Table 2:** Association of demographic characteristics of participants and severity of varicose vein symptoms (*n* = 305).

Characteristics	Pain	Appearance	Activities	Complications
Sex	−0.044	0.011	−0.054	−0.066
Age range	−0.065	0.034	−0.014	−0.021
Education	−0.032	−0.017	−0.036	-.139^a^
Employment	0.005	−0.034	−0.058	−0.006
Occupations	−0.011	0.022	−0.059	0.045

The Pearson test was employed.

^a^ Correlation is significant at 0.05 level (2-tailed).

### Patient preferences for varicose vein treatments

**Figure 2:**
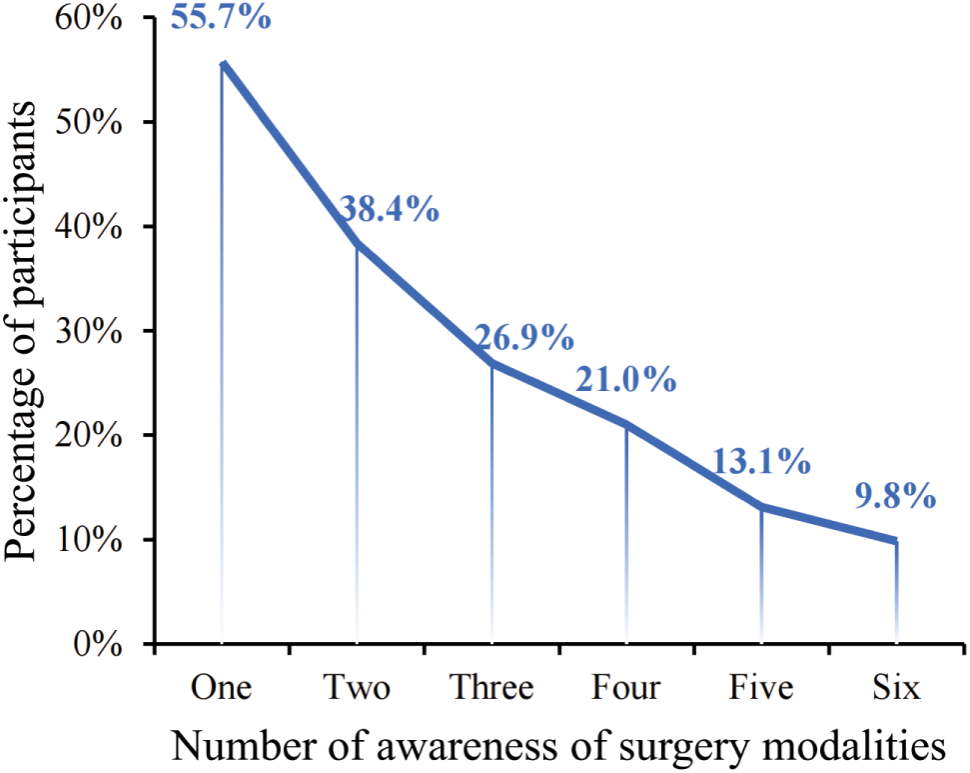
Participants’ awareness of surgery modalities. The surgery modalities including traditional surgery, endovenous laser ablation, radiofrequency ablation, endovenous electrocoagulation, endovenous mechanochemical ablation, ultrasound-guided foam sclerotherapy.

Nearly half of the participants (44.3%, 135/305) lacked information on any surgical options, whereas a slight majority (55.7%, 170/305) possessed familiarity with at least one treatment modality. Within this informed group, 9.8% knew of all six treatment methods presented (Figure 2). Meanwhile, 45.5% (139/305) of the surveyed patients were informed about traditional surgical options, yet awareness was at most 25.5% for any MIT. This suggests a need for more participant knowledge regarding MIT options. Patient surveys discerned that the majority (209/305, 68.5%) declared an inadequate grasp of treatment methodologies to articulate a treatment preference.

Of the 96 patients who delineated a treatment preference, 24.0% (23/96) chose TS, while 76.0% (73/96) favored MIT alternatives. A systematic analysis was undertaken to determine the effect of various participant characteristics on their treatment choice. The findings indicated a higher preference for MIT among male patients compared to female patients (*p* = 0.006), while no significant differences were observed in other demographic features (*p* > 0.05) (Table 3).

**Table 3:** Characteristics of patients with choose preferred treatment options for varicose veins (*n* = 96).

Characteristics	No. of patients choose TS (%) (*n* = 23)	No. of patients choose MIT (%) (*n* = 73)	*P*-value
Sex			0.006^a^
Men	8 (34.8)	49 (67.1)	
Women	15 (65.2)	24 (32.9)	
Age range (Years)			0.865
≤30	4 (17.4)	9 (12.3)	
31–40	13 (56.5)	51 (69.9)	
41–50	5 (21.7)	8 (11.0)	
51–60	1 (4.3)	3 (4.1)	
>60	0 (0)	2 (2.7)	
Education			0.413
Master's degree/PhD	1 (4.3)	9 (12.3)	
Bachelor's degree	14 (60.9)	37 (50.7)	
High school/technical secondary school	1 (4.3)	14 (19.2)	
Junior middle school	3 (13.0)	11 (15.1)	
Below junior middle school	4 (17.4)	2 (2.7)	
Employment			0.703
Awaiting job assignment	2 (8.7)	6 (8.2)	
Part-time	0 (0)	2 (2.7)	
Full time	19 (82.6)	60 (82.2)	
Retire	2 (8.7)	5 (6.8)	
Occupations			0.221
Farmers	1 (4.3)	6 (8.2)	
Public sector employee	2 (8.7)	6 (8.2)	
Private-sector employees	9 (39.1)	11 (15.1)	
Medical staff	8 (34.8)	33 (45.2)	
Self-employed workers	2 (8.7)	13 (17.8)	
No occupation	1 (4.3)	4 (5.5)	

TS, traditional surgical; MIT, minimally invasive treatment.

^a^ Mann-Whitney Chi-Square test.

### Factors influencing patient treatment choices

Concerning the factors affecting patient treatment preferences, in a sample of 305 study participants, those factors rated as “Likely to influence my decision” or “It would definitely influence my decision” by over 50% include “Recommendation of a Vascular Surgeon,” “Type of anesthetic,” and “Number of visits required,” with percentages of 76.4%, 52.7%, and 74.1%, respectively. Spearman's rank correlation method was analyzed to investigate the association between various factors and patients’ treatment preferences. Results evidenced the patients’ preferred treatment options for VVs were significantly affected by vascular surgeon recommendations and the number of follow-up visits (r = 0.129, *p* = 0.024; r = 0.122, *p* = 0.033). Further details can be found in Table 4.

**Table 4:** Factors influencing the choice of patient treatment modality and the relationship with the preferred regimen (*n* = 305).

	Not influence my decision	May influence my decision	Likely to influence my decision	It would definitely influence my decision	Relation to the preferred scheme	
					r	*P*-value
Recommendation of GP	75 (24.6%)	100 (32.8%)	79 (25.9%)	51 (16.7%)	0.018	0.748
Recommendation of a Vascular Surgeon	26 (8.5%)	46 (15.1%)	123 (40.3%)	110 (36.1%)	0.129*	0.024
Previous personal experience	73 (23.9%)	98 (32.1%)	96 (31.5%)	38 (12.5%)	0.028	0.631
Experience of friends or relatives	75 (24.6%)	99 (32.5%)	102 (33.4%)	29 (9.5%)	0.028	0.628
Recovery time off work	70 (23.0%)	95 (31.1%)	89 (29.2%)	51 (16.7%)	−0.011	0.852
Type of anesthetic	74 (24.3%)	70 (23.0%)	127 (41.6%)	34 (11.1%)	0.022	0.699
Number of visits required	31 (10.2%)	48 (15.7%)	80 (26.2%)	146 (47.9%)	0.122*	0.033
Information from a magazine	74 (25.0%)	90 (29.5%)	112 (36.7%)	29 (9.5%)	0.026	0.646
Information from the Internet	80 (26.2%)	106 (34.8%)	95 (31.1%)	24 (7.9%)	−0.008	0.884

The preferred options are classified into traditional surgery, minimally invasive surgery, and unknown options. Correlations were tested using Spearman correlation. GP, general practitioner; MIT, minimally invasive treatment modalities; AUC, area under the curve; LS, least squares; NE, not estimable.

* *P* < 0.05.

**Figure 3:**
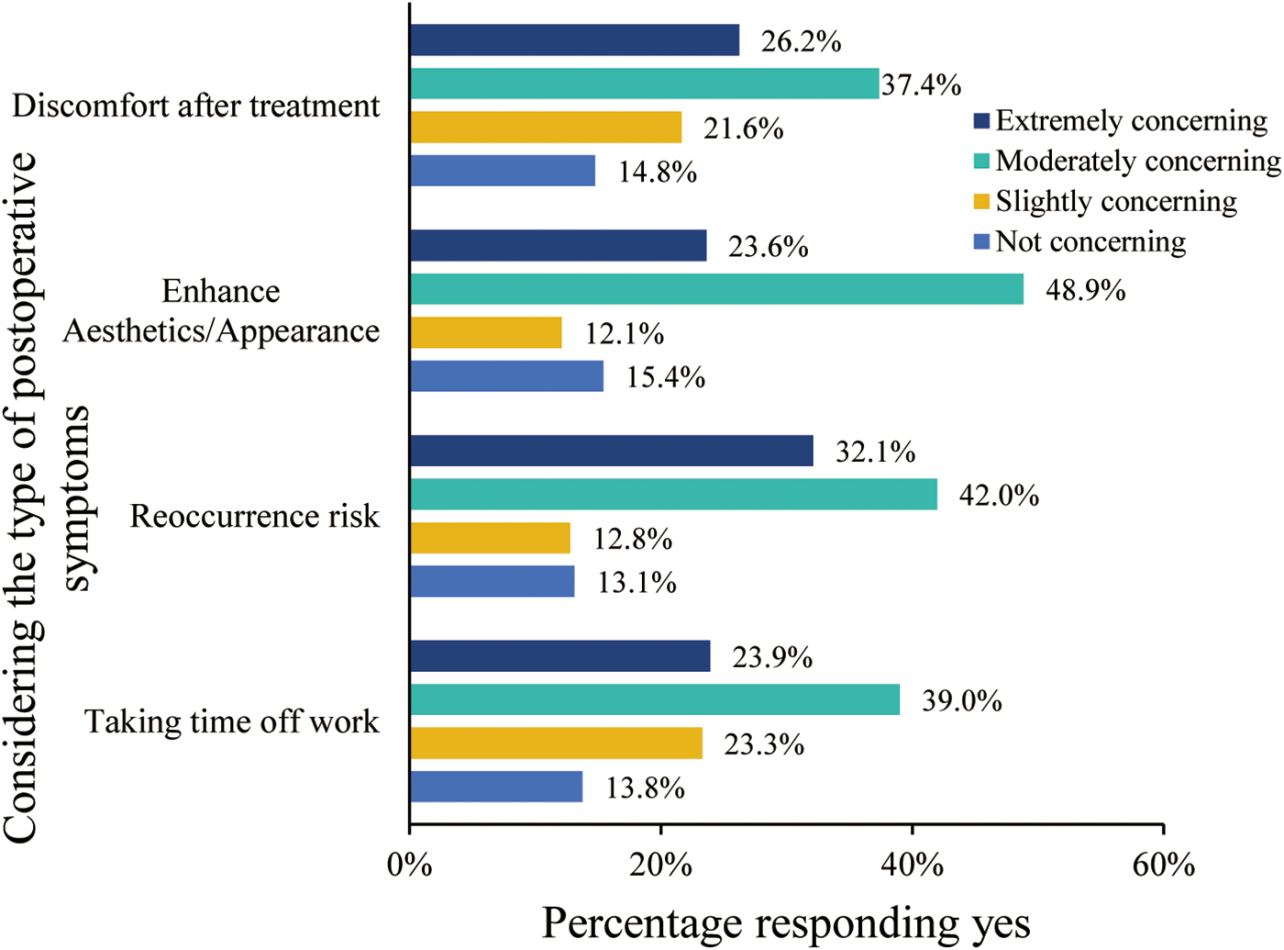
Concerns and preferences of treatment outcomes at choice of treatment modality.

**Figure 4:**
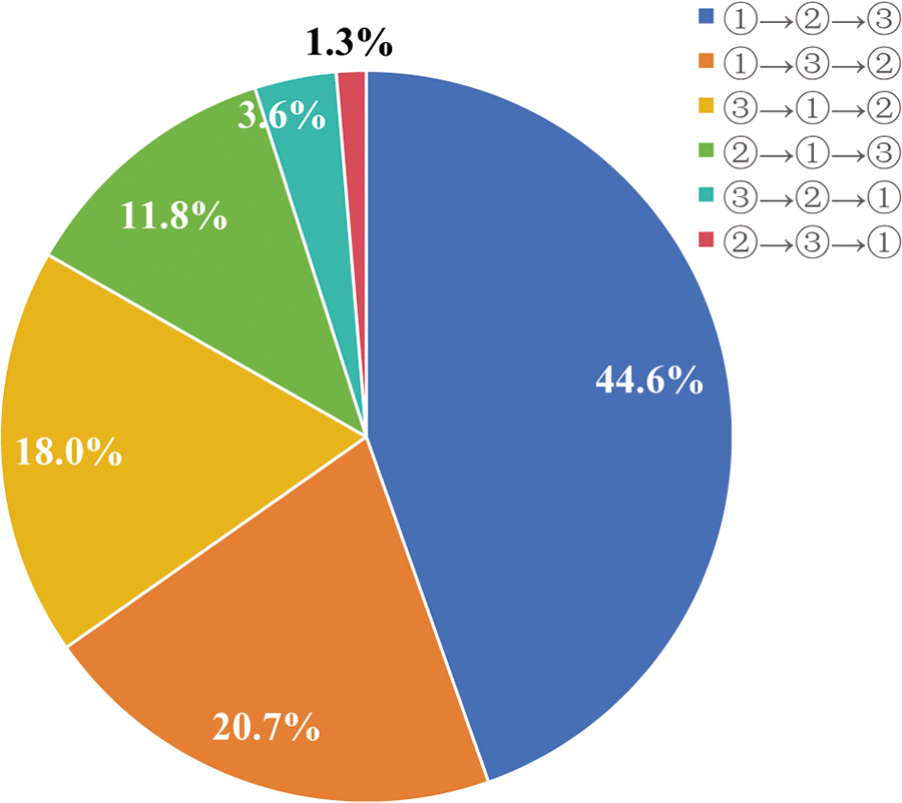
The participants ranked the significance of treatment outcomes when choosing a treatment modality. ① represent alleviate physical symptoms; ② represent enhance aesthetics/appearance; ③ represent reduce reoccurrence risk.

As for the significance of treatment outcomes from the patient's perspective, within the participating 305 individuals, those factors rated as “Moderately concerning” or “Extremely concerning” by over 50% include “Taking time off work”, “Reoccurrence risk”, “Enhance Aesthetics/Appearance”, and “Discomfort after treatment” with percentages of 63.0%,74.1%, 72.5%, and 63.6%, respectively (Figure 3). Notably, upon requesting participants to prioritize the significance of ameliorating physical symptoms, augmenting appearance, and mitigating the risk of varicose vein-related complications, 44.6% sequenced their preferences as: alleviating physical symptoms foremost, followed by enhancing appearance, and subsequently diminishing complication risks (Figure 4).

### Impact of education level on decision-making in varicose vein treatment

Statistical analysis from this research emphasized the significance of education level in the decision-making process for varicose vein treatment options (Table 5). Notably, significance testing indicated that individuals with higher educational attainment were more likely to adhere to GP's advice (*χ*2^ ^= 17.098, *P* = 0.002). Although the educational levels of participants did not appear to show significant differences in the impact of vascular surgeons’ recommendations, the overall influence of these recommendations was high across all levels of education. Moreover, the individual prior experiences of patients with treatment decisions were notably impacted by their educational levels, with those receiving higher education scoring considerably higher on average than their less educated counterparts (*χ*^2 ^= 14.097, *P* = 0.007). Likewise, the experiences of friends or relatives exhibited significant differences among participants with varying educational levels (*χ*^2 ^= 10.639, *P* = 0.031). The focus on recovery time during holidays was significantly influenced by education level, with participants with higher education levels placing greater emphasis on it (*χ*^2 ^= 16.362, *P* = 0.003). Concerning anesthesia type selection, we noted a reverse pattern, where individuals with higher educational levels were less swayed by this aspect; in contrast, those with junior high or lower education tended to base their decisions on the anesthesia type (*χ*^2 ^= 11.449, *P* = 0.022). The highly educated group is more likely to be influenced by magazine information, whereas the low-educated group has lower average scores (*χ*^2 ^= 14.71, *P* = 0.005). For the influence of internet information, participants with junior high school education or below show higher acceptance, as reflected in their higher average scores (*χ*^2 ^= 10.758, *P* = 0.029).

**Table 5:** Factors influencing the choice of patient treatment modality in different educational degrees of participants.

	Mean score of influencing severity scales					X^2^	*P* -value
	Master's degree/PhD	Bachelor's degree	High school/technical secondary school	Junior middle school	Below junior middle school		
Recommendation of GP	2.61 ± 0.98	2.52 ± 1.01	2.06 ± 1.02	2.06 ± 1.02	2.71 ± 0.91	17.098	0.002
Recommendation of a Vascular Surgeon	3.11 ± 0.96	3.13 ± 0.86	2.85 ± 0.99	3.1 ± 0.91	2.79 ± 1.19	4.768	0.312
Previous personal experience	2.83 ± 0.86	2.45 ± 0.97	2.11 ± 1.00	2.06 ± 0.87	2.36 ± 1.01	14.097	0.007
Experience of friends or relatives	2.56 ± 0.86	2.37 ± 0.92	2.1 ± 1.00	2.06 ± 0.87	2.64 ± 1.01	10.639	0.031
Recovery time off work	2.67 ± 1.03	2.58 ± 0.99	2.03 ± 1.02	2.28 ± 0.99	2.36 ± 0.93	16.362	0.003
Type of anesthetic	2.44 ± 1.04	2.58 ± 0.94	2.14 ± 1.00	2.22 ± 0.97	2.36 ± 0.84	11.449	0.022
Number of visits required	2.94 ± 1.06	3.17 ± 0.97	3.07 ± 1.09	3.06 ± 1.10	3.29 ± 0.83	1.056	0.901
Information from a magazine	2.5 ± 0.62	2.46 ± 0.92	2.03 ± 1.01	2.12 ± 0.94	2.64 ± 0.84	14.71	0.005
Information from the Internet	2.33 ± 0.97	2.36 ± 0.91	2.04 ± 0.98	2.02 ± 0.84	1.86 ± 0.66	10.758	0.029

Nonparametric Kruskal–Wallis test was used to test for differences among educational degrees groups.

## Discussion

In Europe, North America, South Korea, and Nepal, MIT has primarily replaced traditional surgery modalities and is recommended as the primary treatment modality for symptomatic VVs (23–25). In the last ten years, MIT has progressively made its way into China. Although the “2014 Chinese Expert Consensus on Diagnosis and Treatment of Chronic Lower Limb Venous Diseases (6)” and the “2019 Chinese Guidelines on Diagnosis and Treatment of Chronic Venous Diseases (7)” provide detailed descriptions of MIT, awareness among Chinese patients with VVs is limited. According to our survey, 47.1% of patients did not know any surgical alternatives, and only 45.1% were familiar with traditional surgery; a mere 10.2% were informed about all six types of surgeries. A survey was conducted among patients with VVs at the vascular surgery outpatient department of a tertiary hospital in China. It was discovered that 47.95% of the patients had a cognition in VVs score of zero (26). A 2010 survey conducted at Charing Cross Hospital, London, showed that 89% of patients were aware of surgery, while less than 40% were aware of endovenous techniques (20). However, these results were also higher than those of our survey. Non-medical professional patients find it very challenging to understand the guidelines of the healthcare industry. These results demonstrate that more knowledge about VVs is needed in China. There is a need for efforts to popularize and promote the transformation of complex and obscure professional knowledge into easily understandable information.

Potential explanations for these outcomes might include: (1) a small proportion of the patients in this study had previously undergone treatment for VVs (7.5%); (2) vascular physicians in China may exhibit a preference for conventional surgeries over MIT (11); (3) traditional varicose vein operations are covered by medical insurance in China, unlike MIT procedures, which could influence physicians’ treatment decisions based on patient insurance coverage (11).

Our study investigated the factors that affect patients’ choices of treatment modalities. The findings indicate that the advice of vascular surgeons and the number of follow-up visits are principal influencers of patient decision-making. Other factors, such as recommendations from general practitioners, personal treatment experiences, and the experiences of friends or relatives, have less influence on patient decisions. These outcomes hint that within clinical practice, the guidance of vascular surgeons and the count of follow-up visits significantly steer patient choices. Comparable studies have also revealed that in managing chronic venous diseases, particularly VVs, the treatment advice of vascular surgeons occupies an essential role (20). The management of chronic conditions demands that experts with extensive professional knowledge and clinical practice craft personalized treatment plans and pay attention to patients’ attitudes and behaviors throughout ongoing treatment. Specialists have garnered valuable professional knowledge and experience through extensive education and practice, enabling them to offer scientific and sensible treatment plans for patients with complex and changing conditions (27).

The number of follow-up visits for chronic diseases has multifaceted psychological impacts on patients. Regular follow-ups can impose financial stress, particularly on patients in weaker economic situations (28); disrupt social engagement, causing patients to retreat from their social spheres steadily (29); and create psychological strain, which could impact the quality of life and the effectiveness of the treatment (30, 31). On the other hand, follow-up visits are crucial for patients to receive medical information and support, which can improve their understanding of the disease and their ability to manage it. When developing a treatment plan, doctors should consider these factors and provide comprehensive and personalized treatment advice addressing the psychological impact of follow-up visits.

Concerning treatment outcomes prioritized by patients, participants believe that safety, effectiveness, improved appearance/cosmetic results, and pain/discomfort during treatment are the most crucial. Interestingly, when participants were asked to prioritize treatment outcomes, 44.6% of them ranked the order of importance as follows: alleviating physical symptoms, improving aesthetic appeal, and minimizing the risk of VVs-related complications. These findings highlight a discrepancy between the treatment preferences expressed by participants and their underlying expectations. It is important for specialists to thoroughly understand each patient's case, uncover the disease's etiologies, identify optimal treatments, and educate patients about their condition. This approach helps patients better understand their health status and improves their adherence to the treatment plan. In addition, specialists can provide psychological support, help patients develop a positive outlook, and reinforce adherence to the treatment protocol. Shared Decision Making (SDM) within Vascular Surgery is consistently promoted as the communication mode of choice during doctor-patient engagements. In contrast with conventional methods, SDM champions the idea of patients playing a more active and proactive role in the decision-making process when considering divergent treatment paths, with clinicians assuming a more advisory capacity (32).

Various studies have shown a distinct preference and acceptance amongst varicose vein patients for MIT (20, 33–35), findings that are echoed in this research. It is thought that patient contentment after traditional surgery modalities for VVs is less favorable, given that such interventions necessitate lengthy incisions and stripping of afflicted veins, which contribute to extended hospitalization, as well as pain and scarring post-surgery (36). This discovery aligns with the prevailing trends in healthcare practice, transitioning to less invasive treatments, boasting quicker recovery periods, and yielding superior cosmetic results (24, 37). Guidance from the NHS endorses the employment of MIT for VVs, a stance bolstered by a Cochrane review that acknowledges treatments like ultrasound-guided foam sclerotherapy, radiofrequency ablation, and endovenous laser therapy as being, at a minimum, equally efficacious as traditional surgery modalities for significant saphenous vein varicosities (38). Lastly, NICE guidelines suggest that endovenous treatments should be considered the first-line therapy for patients diagnosed with VVs and persistent reflux (38, 39).

It is important to acknowledge the limitations of this study when interpreting the findings. The primary limitation is the relatively small sample size and the fact that it was conducted at a single research center. Additionally, the distribution method of the questionnaires may have inadvertently influenced the authenticity of the responses. Although efforts were made to ensure candid responses through anonymous questionnaires and a comfortable environment, it is possible that patients’ answers were unconsciously influenced by a desire to please the authors. Moreover, while the inclusion criteria excluded patients with only telangiectatic or reticular veins and active leg ulceration, no stratified sampling investigation was conducted on varicose veins’ total length and size.

## Conclusion

The study highlights the importance of improving patient education and awareness regarding MIT for VVs in China. It reveals that patients generally prefer MIT due to its less invasive nature, quicker recovery, and better cosmetic outcomes. The guidance provided by vascular surgeons significantly influences treatment choices, emphasizing the need for informed discussions and Shared Decision Making (SDM) in clinical practice. Healthcare providers should customize treatment advice based on individual patient needs, prioritize safety and efficacy, consider the psychological impact of follow-up visits, and enhance patient understanding to optimize the management of varicose veins and ensure patient satisfaction. The preference for MIT suggests its potential as the preferred treatment, aligning with global trends towards less invasive therapies for varicose veins.

## Data Availability

The raw data supporting the conclusions of this article will be made available by the authors, without undue reservation.
