# Supplementary material for: Patient perceptions and preferences of minimally invasive treatment modalities in varicose veins: a cross-sectional survey
Source: Front Cardiovasc Med. 2024 Apr 25;11:1382764. doi: 10.3389/fcvm.2024.1382764 (PMC11079230; doi:10.3389/fcvm.2024.1382764)
Supplement: Supplementary file 1 [file Datasheet1.docx]

# Survey on Patient Preferences Regarding Varicose Veins

## Part A: General characteristics

We appreciate your participation! This brief survey is anonymous and will not be linked to your work record. Please refer to the basic information and chooses the best option from among multiple choice.

| 1. Gender | Male □; Female □ |
| --- | --- |
| 2. Age | _______years |
| 3. Occupations | Farmers □; Medical staff □; Private-sector employees □; Self-employed workers □;  Public sector (included civil servants, teachers, police, military) □;  No occupation (included students, homemakers, retired, and unemployed residents) □ |
| 4. Lever of instruction | Master's degree/PhD □; Bachelor’s degree □; High school / technical secondary school □;  Junior middle school □; Below junior middle school □ |
| 5. Do you work full time or part time? | Full time □; Part time □; Retire□; awaiting job assignment □ |
| 6. Please state your previous history of chronic diseases | Diabetes □; Previous heart attack / heart failure / angina □; Chronic breathing problems □;  Asthma □; Epilepsy □; High blood pressure □; Previous blood clots in the leg or lung □;  Previous stroke or mini stroke □ |

## Part B: The severity of varicose veins and past treatment experience

| 7. Kindly indicate the extent to which the following symptoms of varicose veins have affected you (Please circle one option per row). | | | | |
| --- | --- | --- | --- | --- |
| Symptom | Not affected | Slightly affected | Moderately affected | Severely affected |
| Pain/ache/physical discomfort | 0 | 1 | 2 | 3 |
| Appearance (such as large, unsightly dilated and tortuous veins of the lower extremities, Pigmentation or eczema) | 0 | 1 | 2 | 3 |
| Activity limitations | 0 | 1 | 2 | 3 |
| The risk of the problem reoccurrence | 0 | 1 | 2 | 3 |
| 8. Which of the treatments listed below for varicose veins are you aware of? | | | | |
| Treatment Modalities | YES | | NO | |
| Traditional surgery | □ | | □ | |
| Endovenous laser ablation | □ | | □ | |
| Radiofrequency ablation | □ | | □ | |
| Endovenous electrocoagulation | □ | | □ | |
| Endovenous mechanochemical ablation | □ | | □ | |
| Ultrasound-guided foam sclerotherapy | □ | | □ | |
| Other (please state) | | | | |
| 9. Have you received treatment for varicose veins before? YES □; NO □ | | | | |
| If you answered “yes”, please respond to the question 11 and 12; If you answered “ no”, please skip the question 11 and12, proceed to answer the subsequent questions.  10. Have you been treated with varicose veins before?  Compression therapy□; Drug Therapy □; Ultrasound-guided foam sclerotherapy □; Traditional surgery □; Other □  11. How satisfied are you with the methods used for previous varicose vein treatments?  Dissatisfied □; General □; Satisfied □; Very satisfied□ | | | | |

## Part C: Participant - preference measures

| 12. Which would be your preferred treatment option? | | | | | | |
| --- | --- | --- | --- | --- | --- | --- |
| Treatment Modalities | YES | | | | NO | |
| Traditional surgery | □ | | | | □ | |
| Endovenous laser ablation | □ | | | | □ | |
| Radiofrequency ablation | □ | | | | □ | |
| Endovenous electrocoagulation | □ | | | | □ | |
| Endovenous mechanochemical ablation | □ | | | | □ | |
| Ultrasound-guided foam sclerotherapy | □ | | | | □ | |
| Do not know enough to make a decision (Please give brief reasons): | | | | | | |
| 13. Which things would influence your decision about your choice of treatment? (please circle one option per row) | | | | | | |
|  | | No influence on my decision | May influence my decision | Likely to influence my decision | | Would definitely influence my decision |
| Recommendation of GP | | 0 | 1 | 2 | | 3 |
| Recommendation of a Vascular Surgeon | | 0 | 1 | 2 | | 3 |
| Previous personal experience | | 0 | 1 | 2 | | 3 |
| Experience of friends or relatives | | 0 | 1 | 2 | | 3 |
| Recovery time off work | | 0 | 1 | 2 | | 3 |
| Type of anaesthetic | | 0 | 1 | 2 | | 3 |
| Number of visits required | | 0 | 1 | 2 | | 3 |
| What you have read in a magazine | | 0 | 1 | 2 | | 3 |
| What you have read on the Internet | | 0 | 1 | 2 | | 3 |
| Comments: | | | | | | |
| 14. When considering treatment for your varicose veins which of the following concerns you? (please circle one option per row) | | | | | | |
| Symptom | | Not concerning | Slightly concerning | Moderately concerning | | Extremely concerning |
| Taking time off work | |  |  |  | |  |
| Reoccurrence risk | |  |  |  | |  |
| Enhance Aesthetics/Appearance | |  |  |  | |  |
| Discomfort after treatment | |  |  |  | |  |
| 15. Which outcome following treatment is most important to you(please rank:1=most important 3 =least important) | | | | | | |
| □ Resolution of physical symptoms e.g.pain,aching;  □ Improved cosmetic appearance;  □ Reduced risk of complications related to varicose veins | | | | | | |
